# Synanthropic rodents and their ectoparasites as carriers of a novel haemoplasma and vector-borne, zoonotic pathogens indoors

**DOI:** 10.1186/s13071-014-0630-3

**Published:** 2015-01-15

**Authors:** Sándor Hornok, Gábor Földvári, Krisztina Rigó, Marina L Meli, Enikő Gönczi, Attila Répási, Róbert Farkas, Ibolya Papp, Jenő Kontschán, Regina Hofmann-Lehmann

**Affiliations:** Department of Parasitology and Zoology, Faculty of Veterinary Science, Szent István University, Budapest, Hungary; Clinical Laboratory and Center for Clinical Studies, Vetsuisse Faculty, University of Zurich, Zurich, Switzerland; County Veterinary Station, Borsod-Abaúj-Zemplén, Miskolc, Hungary; Veterinary Clinic, Mohács, Hungary; Plant Protection Institute, Centre of Agricultural Research of Hungarian Academy of Sciences, Budapest, Hungary

**Keywords:** Mouse, Rat, *Rickettsia*, *Anaplasma*, *Borrelia*, *Bartonella*, Haemoplasma

## Abstract

**Background:**

Despite their close association with human dwellings, the role of synanthropic rodents in the epidemiology of vector-borne infections is seldom studied. The aim of the present study was to compensate for this lack of information, by the molecular investigation of vector-borne bacteria in peridomestic rodents and their ectoparasites.

**Findings:**

Fifty-two rodents (mainly house mice and brown rats) were caught alive in buildings and checked for blood-sucking ectoparasites; followed by molecular analysis of these, together with spleen samples, for the presence of vector-borne agents. Haemoplasma infection was significantly more prevalent among brown rats, than among house mice. A novel haemoplasma genotype (with only 92-93% similarity to *Candidatus* Mycoplasma turicensis and *M. coccoides* in its 16S rRNA gene) was detected in a harvest mouse and a brown rat. Sporadic occurrence of *Rickettsia helvetica*, *Anaplasma phagocytophilum*, *Borrelia burgdorferi* s.l. and *Bartonella* sp. was also noted in rodents and/or their ectoparasites.

**Conclusions:**

These results indicate that synanthropic rodents, although with low prevalence, may carry zoonotic and vector-borne pathogens indoors.

## Findings

### Background

Owing to the number of their species, mice and rats belong to the largest family (Muridae) and order (Rodentia) of mammals. Most rodent species may reach large individual numbers in a wide range of habitats, justifying their epidemiological significance as reservoirs and hosts of pathogens. Among these pathogens, particularly those have received scientific attention during the past few years, which are transmitted by blood-sucking arthropod vectors [1-3].

Ticks are regarded as the most important vectors of disease agents in the temperate zone [4]. However, synanthropic rodents, such as the house mouse (*Mus musculus*) and the brown rat (*Rattus norvegicus*), which live in or near human dwellings and animal keeping facilities, are usually not tick-infested [2]. Consequently, they are considered to be less important reservoirs of tick-borne pathogens, and seldom evaluated in this respect. Similarly, some hemisynanthropic rodents (living on crops), such as the harvest mouse (*Micromys minutus*), also less frequently harbor ticks, although they are partly sympatric with more frequently tick-infested rodent species [2]. Nevertheless, even these (hemi)synanthropic rodents are known to have blood-sucking ectoparasites, such as fleas or mites [1,5], which are not host-specific and thus may suck blood on humans e.g. if their primary host dies [6,7]. However, information on the pathogens carried by ectoparasites of synanthropic rodents is scarce, partly because during relevant surveys house mice and brown rats are usually caught with traps that kill them and will cause loss of ectoparasites before they could be collected for molecular analyses.

Taken together, ectoparasite communities and associated (zoonotic) pathogens of various wildlife rodent species represent a well-studied issue [8,9], but in this context much less is known about synanthropic rodents (although these could be more relevant to assess from the point of view of zoonotic vector-borne pathogens). Therefore, the present study was undertaken to elucidate if (hemi)synanthropic rodents and their blood-sucking ectoparasites are infected with five important categories of vector-borne pathogens (haemoplasmas, rickettsiae, Anaplasmataceae, *Borrelia burgdorferi* sensu lato [s.l.] and bartonellae), all including zoonotic agents.

### Methods

#### Sample collection

In the present study 52 rodents were collected on ten locations near the north-eastern and southern border of Hungary in 2011. Live catch traps for mice and rats (Nortene, Capital Gardens Ltd., England) were installed in farm buildings and houses. During anaesthesia all small mammals were scrutinized for the presence of ectoparasites which were removed with flea combs or brushes onto white paper. After euthanasia a fraction of the spleen was removed and stored frozen at −20°C.

Fleas and lice were identified on the species level according to standard morphological keys. Mites were treated with lactic acid for transparency and their genera/species determined according to [10]. Ectoparasites of the same species collected from the same host were stored (in 70% ethanol) and processed together.

#### Molecular analyses

DNA was extracted individually or in pools (Table 1) according to [11]. The quality and quantity of DNA was assessed by an 18S rRNA gene TaqMan real-time PCR [12]. Molecular analyses for rickettsiae, *A. phagocytophilum*, *Borrelia burgdorferi* s.l. and bartonellae were performed with TaqMan real-time PCRs as reported [12-15]. The presence of Anaplasmataceae was evaluated with the methods described in [16].

**Table 1:** **Pathogens and endosymbionts detected in the spleen and ectoparasites of synanthropic and hemisynanthropic rodents**

**Host species**	**Sample type**	**Number of specimens per animal**	**Total number of samples analysed**	**PCR positives/all tested**						
				**Haemotropic** ***Mycoplasma*** **spp.**		**Rickettsiales**			**Others**	
				**Haemofelis group**	**Result of sequencing**	***Rickettsia helvetica***	***Anaplasma phagocytophilum***	***Wolbachia*** **sp.**	***Borrelia burgdorferi*** **s.l.**	***Bartonella*** **sp.**
*Mus musculus*	**spleen**	1	37	21/37	not successful	1/37			1/37	
	**flea** (*Leptopsylla segnis*)	1-3	10		-	2/10		1/10		1/10
	**mite** (*Laelaps algericus*)	1-15	3		-	1/3	1/3			
*Micromys minutus*	**spleen**	1	1	1/1	nM. sp. (1×, 100%)					
*Rattus norvegicus*	**spleen**	1	14	13/14	nM. sp. (1×, 98%), M. sp. [AB752303] (1×, 99%)*				1/14	
	**louse** (*Polyplax spinulosa*)	4-50	2	1*/2	-					

Abbreviation: (n)M. sp. - (novel) *Mycoplasma* species.

One pool contained all ectoparasites of the same species removed from one host individual. All samples were tested for all pathogens shown, but only positive results are indicated. PCR positivity of corresponding samples (ectoparasites and their host) are marked with asterisk (*).

General screening for haemoplasma DNA was done with a SYBR Green real-time PCR [17], and positive samples were then tested with two haemoplasma group-specific TaqMan real-time PCRs (haemofelis vs. haemominutum groups: [18]). From all samples, yielding threshold cycle (Ct) values below 30 in the group-specific assay, cloning and sequencing was attempted repeatedly as reported [19]. New sequences were submitted to the GenBank.

All PCRs were run while including appropriate positive and negative controls. Phylogenetic analysis was performed with the MEGA 6.06 program by using the neighbor-joining method with maximum composite likelihood model and 1000 bootstrap resamplings [20].

#### Statistical analysis

Prevalence data were compared with Fisher’s exact test [21], and differences were considered significant when P < 0.05.

#### Ethical approval

Animals were captured, handled and euthanized while observing the regulations on animal welfare (28/1998).

### Results and discussion

Altogether 51 synanthropic rodents were collected (37 house mice and 14 brown rats), as well as one individual of a hemisynanthropic species (harvest mouse). Their ectoparasites included fleas and mites on ten and three mice, respectively, as well as lice on two rats (Table 1). No ticks were found. The latter result is consistent with previously published data [2], confirming that synanthropic rodents are seldom parasitized by ixodid ticks.

**Figure 1:**
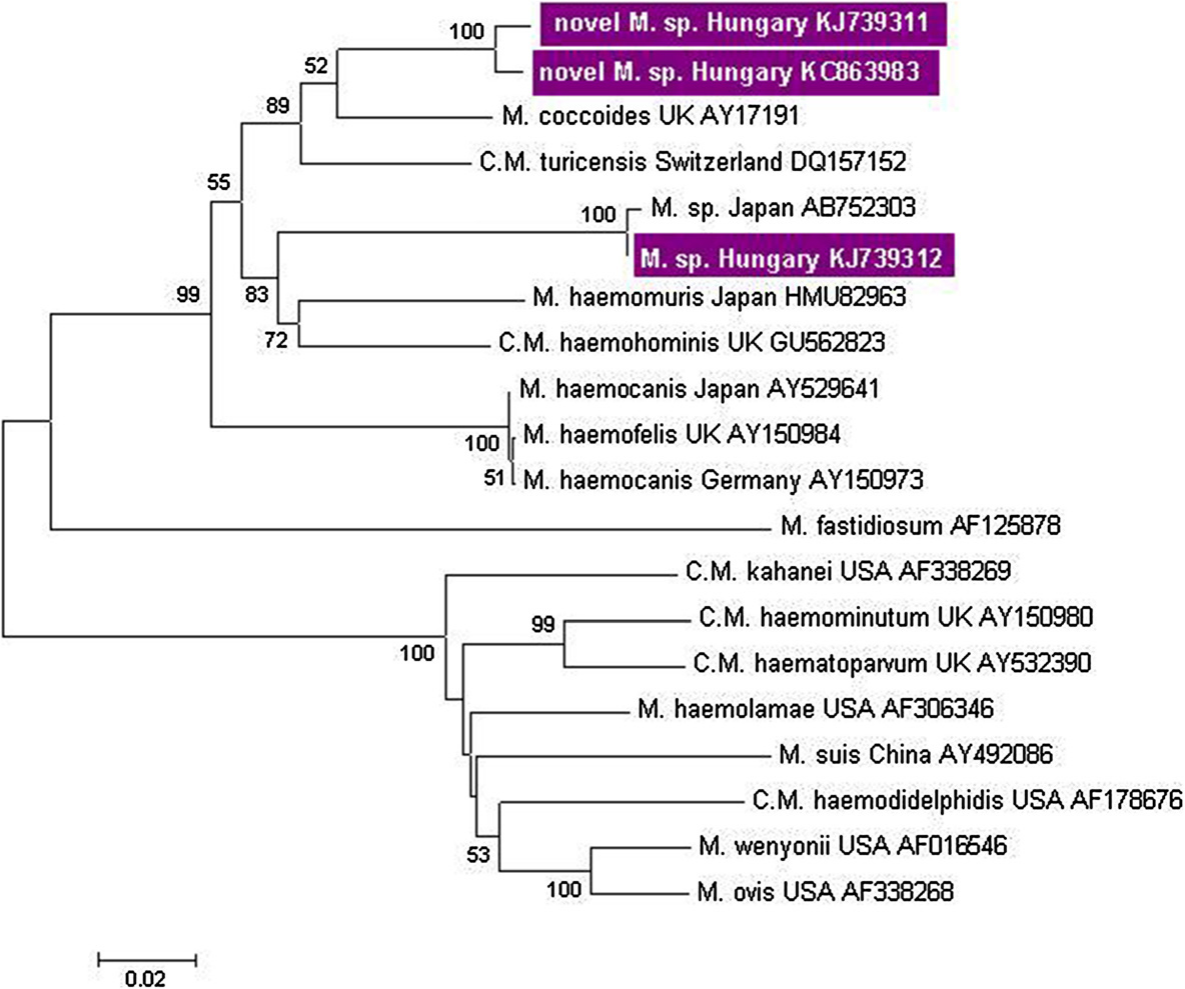
**Phylogenetic relationships of the haemoplasma isolates found in the present study with other species of the category.** Branch lengths correlate to the number of substitutions inferred according to the scale shown.

Results of molecular analyses are shown in Table 1. In the category of haemotropic *Mycoplasma* spp. (haemoplasmas) only members of the haemofelis group were detected. This is not surprising in light of the fact that both known murine haemoplasmas (*M. haemomuris* and *M. coccoides*) represent this category [18]. Haemoplasma infection was significantly (P = 0.019) more prevalent among rats, than mice. Haemoplasmas were detected in blood-sucking lice, but not in mites and fleas of PCR positive rodents, in line with the known vector competence of *Polyplax spinulosa* [22,23]. Sequencing of the PCR product from the harvest mouse spleen DNA sample revealed a novel haemoplasma genotype (KC863983), exhibiting the closest, but only 93% homology to *Candidatus* M. turicensis (DQ157152) and *M. coccoides* (AY171918) (Figure 1). Interestingly, a similar genotype (having 98% sequence homology to the above novel haemoplasma, but only 92% and 93% to *M. coccoides* and *Candidatus* M. turicensis, respectively) was identified in a rat spleen sample (KJ739311). Phylogenetically the two genotypes of this novel haemoplasma represent a separate cluster from both formerly known murine haemoplasmas (Figure 1). In addition, a haemoplasma sequence highly (99%) similar to a Japanese rat *Mycoplasma* sp. isolate (AB752303, [24]) was recovered from another rat (KJ739312, Figure 1). Concerning the potential medical-epidemiological implications of these findings, while rodent haemoplasmas are not known to be zoonotic *per se*, the recently discovered human haemotropic *Mycoplasma* species (*C*. Mycoplasma haemohominis) is genetically most closely related to these haemoplasmas [25] (Figure 1).

In the category of rickettsial agents, *Rickettsia helvetica* was present in one house mouse, and in fleas/mites of three further house mice (Table 1). Although this zoonotic, potentially pathogenic bacterium is known to occur in wildlife rodents and their ectoparasites [26], it was (to the best of our knowledge) hitherto unreported in house mouse and its fleas or mites. No other rickettsiae were found.

*Anaplasma phagocytophilum* was detected in mouse mites (Table 1). This zoonotic pathogen has already been reported from laelapid mites from wild rodents [5], but this appears to be its first finding in *Laelaps algericus* of the house mouse.

The epidemiological significance of these data is provided by the fact that both fleas and mites of rodents are known to feed on man [6,7]. Rodent fleas are competent vectors of rickettsiae other than *R. helvetica* [26], and non-tick acarines can potentially transmit *A. phagocytophilum* [5].

In addition, a sequence (KC852875) with the closest (98%) homology to *Wolbachia* sp. endosymbionts (e.g. AB746399) was identified in the mouse flea (Table 1).

*Borrelia burgdorferi* s.l., including the causative agents of Lyme disease in animals and humans, was also detected in one house mouse and one brown rat (Table 1), but in none of the ectoparasites. These results improve the paucity of data on the occurrence of borreliae in these two rodent species [27,28]. Despite being a principally tick-borne group of pathogens, *B. burgdorferi* s.l. was reported to be potentially transmitted by rodent mites [29]. Correspondingly, it is not unexpected to find PCR-positive rodents in the absence of tick-infestation (as reported here), although previous exposure to ticks in the present case cannot be ruled out.

Finally, *Bartonella* sp. positivity was detected for the first time in the flea *Leptopsylla segnis* from a mouse (Table 1), corroborating previous finding of bartonellae in the same flea species from rats [30]. In this context it is relevant to note that fleas are regarded as competent vectors of *Bartonella* spp. [31], which are known to occur in both wild and urbanized rodents [32,33].

Except for the mutual haemoplasma positivity of one rat and its lice, the PCR status of vectors did not correspond to that of their host (i.e. in most cases PCR positive ectoparasites were found on PCR negative hosts, and *vice versa*). This suggests that the evaluated rodent species or their ectoparasites (albeit with low prevalence) could become prolonged carriers of relevant pathogens, increasing the epidemiological significance of the present findings.

### Conclusions

In summary, genetic data are provided here for the first time on the existence of a novel rodent haemoplasma. Results of the molecular analyses in this study also compensate for the scarcity of information on the epidemiological significance of synanthropic rodents and their blood-sucking ectoparasites. The majority of detected pathogens (*R. helvetica*, *A. phagocytophilum* and *Borrelia burgdorferi* s.l.) are transmitted by ticks, in Europe by *Ixodes ricinus*. Although the present results confirm that synanthropic rodents are seldom tick infested, they or their ectoparasites can still harbor tick-borne, zoonotic pathogens indoors.
